# Traditional and Additional Isokinetic Knee Strength Assessments of Athletes; Post-Operative Results of Hamstring Autograft ACL Reconstruction

**DOI:** 10.3390/medicina58091187

**Published:** 2022-08-31

**Authors:** Ahmet Serhat Genç, Nizamettin Güzel

**Affiliations:** Department of Orthopedics and Traumatology, Samsun Training and Research Hospital, Samsun 55090, Turkey

**Keywords:** anterior cruciate ligament reconstruction, return to sports, isokinetic evaluations, athletes

## Abstract

*Background and Objectives:* Anterior cruciate ligament (ACL) injuries are common injuries in athletes, and, accordingly, ACL reconstruction (ACLR) is one of the most common orthopedic surgical procedures performed on athletes. This study aims to compare the 6-month post-operative isokinetic knee strength evaluations of the semitendinous/gracilis (ST/G) ACLR technique performed on healthy (HK) and ACLR knees of athletes. *Materials and Methods:* A retrospective cohort of 29 athletes from various sports branches who underwent ST/G ACLR technique by the same surgeon were evaluated. The isokinetic knee extension (Ex) and flexion (Flx) strength of the patients on the HK and ACLR sides were evaluated with a series consisting of three different angular velocities (60, 180, and 240°/s). In addition to the traditional evaluations of peak torque (PT) and hamstring/quadriceps (H/Q) parameters, the findings were also evaluated with additional parameters such as the joint angle at peak torque (JAPT), time to peak torque (TPT), and reciprocal delay (RD). *Results:* There was a significant improvement in the mean Lysholm, Tegner, and IKDC scores after surgery compared with preoperative levels (*p* < 0.05). As for the isokinetic PT values, there were significant differences in favor of HK in the 60°/s Flx, 180°, and 240°/s Ex phases (*p* < 0.05). In addition, there was a significant difference in the 60° and 180°/s Flx phases in RD (*p* < 0.05). In H/Q ratio, TPT, and JAPT values, no significant difference was observed between HK and ACLR at all angular velocities. *Conclusions:* The findings showed that the ST/G 6-month post-operative isokinetic knee strength in athletes produced high results in HK, and, when evaluated in terms of returning to sports, the H/Q ratios on the ACLR side were sufficient to make the decision to return to sports. It was found that the ACLR side was slower than the HK side in the reciprocal transitions, particularly in the Flx phase. We believe that this results from the deformation of the hamstring muscle after reconstruction of the ST/G ACLR side.

## 1. Introduction

Anterior cruciate ligament (ACL) structure, which is the most important ligament providing stability to the knee joint, is of great importance since it adjusts the stiffness of the quadriceps (Q) and hamstring (H) muscles in the agonist–antagonist structure of the knee. It also enables safe reciprocal movements such as extension (Ex) and flexion (Flx) and performs a proprioceptive function [1]. ACL, which is of great importance particularly for the athletic population, is considered as one of the most common injuries faced by athletes. When considered in this regard, ACL reconstruction (ACLR) is one of the most common orthopedic surgical procedures performed in sports medicine [2]. ACL injuries typically occur as a result of sudden deceleration, changes in direction, or harsh blows to the knee [3]. Although ACLR is applied in different ways by orthopedists, one of the most commonly applied methods is a conventional one performed with hamstring autograft semitendinosus/gracilis (ST/G) tendons [4].

ACL injuries have many adverse effects on thigh muscle function, including reducing muscle strength, resulting in instability in strength-related torque generation [5,6]. In this regard, it is crucial to evaluate return to sports (RTS) after ACLR, the rehabilitation process and whether the knee strength has reached the optimal level; these evaluations are most objectively provided by isokinetic dynamometers [7,8,9]. Conventionally, in isokinetic dynamometers, the Ex and Flx peak torque (PT) strength applied reciprocally by the knee, as well as the H/Q strength ratios produced by the H and Q muscles at different angular velocities can be evaluated [10]. The H/Q ratio used after ACLR can be defined as unequal strength between the right and left Q and H muscles. This ratio increases as the angular velocity increases in isokinetic dynamometers and can range between 50% and 80% [11]. Ratios of 60–65% at an angular velocity of 60° are considered normal [12]. Although this ratio, which is considered normal, is acceptable, it may reveal different results in athletes depending on the physical requirements and muscle structure of the sports branch. 

For isokinetic dynamometers, researchers generally evaluate healthy athletes and those with ACLR history, using conventional parameters such as PT and H/Q ratios [8,13]. However, thanks to isokinetic dynamometers, not only these traditional data but also many different data such as joint angle at peak torque (JAPT), time to peak torque (TPT) and reciprocal delay (RD), which shows the time loss in reciprocal transitions between Ex and Flx phases, can be obtained. Accordingly, researchers pointed out that muscle reaction times during strength generation play an important role in preventing musculoskeletal injuries, particularly those of the joints [14,15]. The rapid stabilization of the joints by the muscles during sudden and rapid movements suggests that the agonist and antagonist muscles will minimize injuries by doing mutual neuromuscular work. Delays in reaction times of agonist–antagonist muscles can cause serious knee injuries, particularly in athletes who frequently make sudden speed and direction changes. Zabka et al. [16] also made the same point. In light of this information, it is believed that, in addition to traditional parameters, parameters such as JAPT, TPT, and RD are also important for evaluating rehabilitation and RTS periods after ACLR in athletes. However, a review of the literature revealed that there was no study conducted to evaluate JAPT, TPT, and RD parameters in addition to the traditional parameters after ACLR in athletes. In this regard, the findings of our study will contribute to the literature. Our current study is the first study to examine JAPT, TPT, and RD parameters in post-ACLR athletes.

This study aimed to compare the results of PT, H/Q ratio, JAPT, TPT, and RD parameters in healthy (HK) and ACLR knees, as produced by H and Q muscle strength 6-month post-operative ST/G ACLR in athletes. The study was based on the hypothesis that there would be no difference between HK and ACLR knees in athletes regarding the parameters examined.

## 2. Materials and Methods

### 2.1. Participants

Ethics committee approval of the study was granted by Samsun Training and Research Hospital Clinical Research Ethics Committee and the study was conducted in accordance with the Helsinki Declaration. The study was conducted between May 2020 and October 2021 and included a retrospective cohort of athletes (*n* = 29) from various sports branches who underwent the traditional ACL reconstruction (ST/G) technique by the same surgeon (Table 1). An a priori test with GPower 3.1 program was used to determine the number of participants.

The inclusion criteria for the study were as follows: Being a male between the ages of 18 and 35 with isolated ACL rupture in only one knee and without any concomitant meniscus, chondral, or other ligament injury, other neuromuscular or musculoskeletal injury, or a history of contralateral knee surgery or injury. Lysholm, Tegner, and International Knee Documentation Committee (IKDC) scores of the patients were evaluated before and at the 6th month postoperatively [17,18]. To reduce variability in the recovery period, all participants were referred to the same rehabilitation program after surgery.

### 2.2. Semitendinosus/Gracilis Autograft Method

In ST/G ACLR, semitendinosus and gracilis tendon autografts from the same leg are used. Both tendons are folded in half to form a four-strand graft. A closed socket is opened into the femur via the medial arthroscopic portal. An open tunnel is opened from the outside into the tibia. Suspension fixation is used to fix the graft to the femur, and interference screw fixation is used to fix it to the tibia.

### 2.3. Procedures

Lysholm, Tegner, IKDC scores (pre- and post-operative) and 6-month post-operative isokinetic knee Ex and Flx performances of all participants were determined. For these measurements, all participants visited the laboratory 3 times in total in addition to the routine postoperative controls. In the first visit (pre-operative), the participants filled in the subjective questionnaires consisting of Lysholm, Tegner, and IKDC scales and were informed about the study. In the second visit (6 months post-operative), anthropometric data were obtained and isokinetic knee strength tests to be performed in the next visit were experienced by the participants (familiarization). In the third laboratory visit (2 days after the second visit), Lysholm, Tegner, and IKDC scales were filled for the second time (post-operative) and 6-month post-operative isokinetic knee Ex and Flx performances were measured. 

The knee Ex and Flx strength of the participants in the HK and ACLR sides were evaluated with a series consisting of 3 different angular velocities (60, 180, and 240°/s). A computer-controlled isokinetic dynamometer (Humac Norm Testing and Rehabilitation System, CSMI, USA) was used for this evaluation. Immediately after the general warm-up protocol, the seat, dynamometer, adapter, and other settings of the dynamometer were adjusted for the subjects according to the fixed protocol set for knee Ex and Flx strength. According to this protocol, the mobility angle (range of motion (ROM)) of the subjects’ knee joints was taken to the 0–90° position. The back support of the chair was adjusted at the hip joint angle of 85° (0° = full extension). Dynamometer arm rotation was set at the level of the lateral femoral epicondyle. The pad on which the lower leg attachment was fixed was placed proximal to the lateral malleus. The belts used to prevent body and Q muscle movement were tightened with a three-finger gap between the body and the Q muscle, and each subject held the hand grips on both sides of the seat during the test. The ankle was placed on the leg stabilizer under the chair to prevent movement of the contralateral limb. Before all tests, the knee joint rotation axis (lateral femoral condyle) and rotation axes were calibrated on the same line. Before starting the measurements, the torque value of the knee joint produced by the leg at 90° Ex (full extension) in the free position was measured with a dynamometer in all subjects in order to eliminate the effect of gravity. It was ensured that the torque values obtained with the measurements were only strength-based torque values. Before starting the test, all subjects were asked to apply their knee strength at maximum level to achieve a positive test and to obtain maximum results.

Isokinetic knee Ex and Flx strength for both HK and ACLR sides were measured by adjusting the fixed protocol performed with sequential concentric/concentric (Con/Con) contractions at angular velocities of 60°/s (4 repetitions, 15 s rest, 5 retest), 180°/s (4 repetitions, 15 s rest, 5 retest), and 240°/s (4 repetitions, 15 s rest, 15 retest). One-minute rest intervals were given between angular velocities, and 5 min rest intervals were given between ACLR and HK sides. The tests were first applied to the ACLR sides. In order to achieve maximal results, verbal support was given to the subjects throughout the measurements to increase motivation. PT values were recorded in Newton meters (Nm); H/Q ratios were recorded in percentage (%); JAPT values were recorded in degrees (°) and TPT and RD values were recorded in sec.

### 2.4. Statistical Analysis

The SPSS 21 program was used in the statistical analysis of the research. Results were presented as mean and standard deviation. The Shapiro–Wilk test was used as a normality test and Levene’s test was used for homogeneity assumptions. The paired sample test was used to compare paired groups (HK-ACLR and pre-post). In addition, in the comparison of paired groups, effect sizes were found according to Cohen’s d effect size (*M*_2_ − *M*_1_)/*SD*_pooled_). According to this formula, a d value of <0.2 was defined as weak effect size while a d value of 0.5 was defined as moderate and a d value of >0.8 was defined as strong effect size. The statistical results were evaluated within a significance level of *p* < 0.05. 

## 3. Results

Compared to pre-operative levels, there was a significant improvement in the mean Lysholm, Tegner, and IKDC scores at the post-operative level (*p* < 0.05). Lysholm scores were 71.96 ± 16.99 and 98.17 ± 3.45, Tegner scores were 6.48 ± 1.45 and 6.00 ± 1.64, and 50.34 ± 8.58 and 90.97 ± 5.95 for the IKDC subjective scores, pre- and post-operatively, respectively (Table 2).

**Figure 1:**
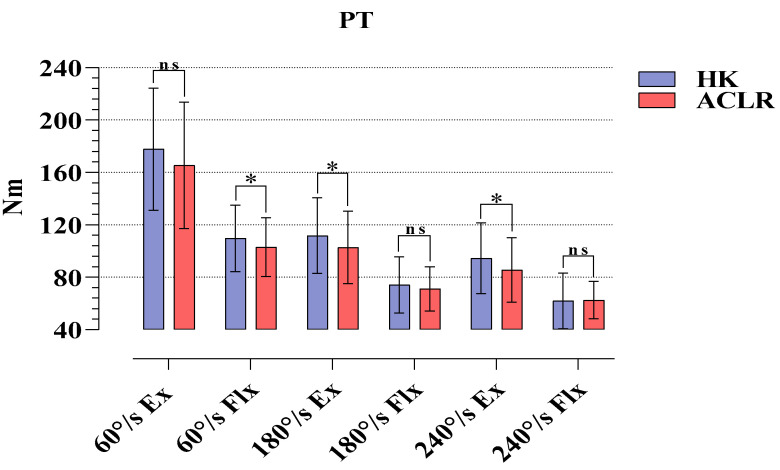
Comparison of PT values obtained in isokinetic tests for HK and ACLR. ns, nonsignificant; * *p* < 0.05; HK, healthy knee; ACLR, anterior cruciate ligament reconstruction knee; Ex, extension; Flx, flexion.

An evaluation of the strength applied by the subjects in the isokinetic tests on the HK and ACLR sides showed that there were statistical significances at 60°/s Flx (*p* = 0.032, %95 CI = 0.63–12.68), 180°/s Ex (*p* = 0.034, %95 CI = 0.72–17.34), and 240°/s Ex (*p* = 0.011, %95 CI = 2.16–15.56). No significance was found in other isokinetic tests (*p* > 0.05) (Figure 1).

**Figure 2:**
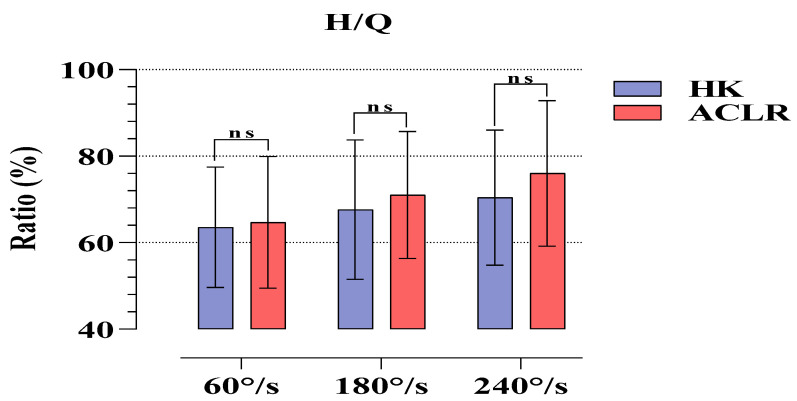
Comparison of H/Q ratios obtained in isokinetic tests for HK and ACLR. ns, nonsignificant; HK, healthy knee; ACLR, anterior cruciate ligament reconstruction knee; H/Q, hamstring/quadriceps.

Figure 2 compares the H/Q ratios revealed by the strength values applied by the subjects in the isokinetic tests on the HK and ACLR sides. No statistical significance was found at any angular velocity (*p* > 0.05).

**Figure 3:**
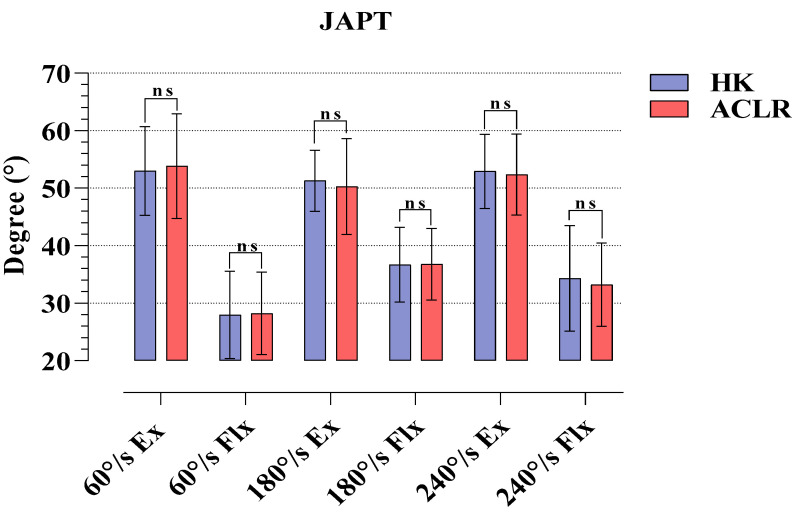
Comparison of JAPT values obtained in isokinetic tests for HK and ACLR. ns, nonsignificant; HK, healthy knee; ACLR, anterior cruciate ligament reconstruction knee.

In Figure 3, the JAPT parameters revealed by the subjects at different angular velocities in the isokinetic tests on the HK and ACLR sides are compared. The results show that there was no statistical significance in the Ex and Flx phases at any angular velocity (*p* > 0.05).

**Figure 4:**
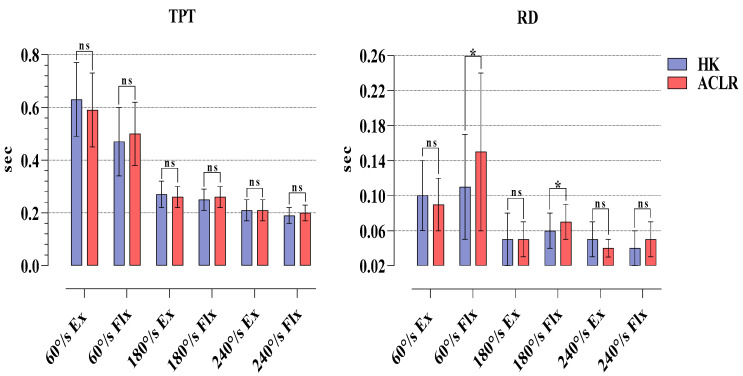
Comparison of TPT and RD values obtained in isokinetic tests for HK and ACLR. ns, nonsignificant; * *p* < 0.05; HK, healthy knee; ACLR, anterior cruciate ligament reconstruction knee; H/Q, hamstring/quadriceps; Ex, extension; Flx, flexion.

The results of isokinetic TPT in the HK and ACLR sides were compared. The results showed that there was no statistical significance at any angular velocity in Ex and Flx phases (*p* > 0.05). In RD, there was a statistical significance only at 60°/s Flx (*p* = 0.024. %95 CI = −0.07/−0.01). There was no statistical significance at other parameters (*p* > 0.05) (Figure 4).

## 4. Discussion

The results of our study are as follows: The six-month post-operative Tegner, Lysholm and IKDC scores of the athletes after ST/G ACLR were significant regarding pre-operative and post-operative findings. Moreover, in terms of PT values, it was found that HK had higher strength in 60°/s Flx, and 180 and 240°/s Ex phases compared to the ACLR side. The H/Q percentages showed that the athletes were within normal ranges at all angular velocities. There was no difference between the two sides in JAPT and TPT. In RD, significant reciprocal delays were observed only in the 60 and 180°/s Flx phases. 

One of the methods which provides the most objective results in terms of revealing the differences between the lower extremities of healthy individuals or individuals with disability or an operation history of lower extremity is using measurements conducted with isokinetic dynamometers. As a matter of fact, researchers frequently use this method after ACLR, which is commonly performed in the athletic population [19,20]. After ST/G (hamstring autograft) operations, a frequently preferred ACLR type for the athletic population, researchers frequently use isokinetic dynamometers to set the RTS duration of the athletes and to reveal the differences in ACLR and HK sides [21,22]. In a retrospective cohort study, it was reported that isokinetic knee strength evaluations of athletes who underwent ST/G and quadricep tendon autograft (QTA) ACLR revealed that those who underwent QTA would recover slowly, particularly in extensor muscle functions, and that return to sports was approximately 1 year. In addition, in the same study, no negative findings were found in functional recovery and RTS durations for ST/G [21]. In another study, it was revealed that the post-operative findings of people who underwent ST/G and QTA ACLR revealed similar findings in both graft types with regard to Ex, Flx, and H/Q ratios. However, the same study also reported that the QTA group had relatively higher H/Q ratios compared to those of the ST/G [23]. Similarly, there are other studies in the literature reporting that QTA had significantly higher H/Q ratios compared to ST/G [24]. These studies were conducted at 60 and 180^o^/s angular velocities. Although the present study focuses only on ST/G ACLR, our findings show that ST/G can be a method of choice to reduce the functional recovery period of the knee and the return to sports duration in athletes. In our study, although there were differences in PT values between the HK and ACLR sides at different angular velocities, it was found that there were similar ratios on both sides regarding H/Q. However, there are studies reporting contrasting results to the above-mentioned studies and to the present study. In a study conducted by Sinding et al. on the 1-year post-operative results of patients who had QTA and ST/G ACLR, it was reported that ST/G caused minor deformation on the functional structures of both extensor and flexor muscles and that there were major deficits in extensors in QTA. However, they did not report a preference for either graft functionally [22]. Other studies comparing QTA and ST/G with Pateller tendon autograft (PTA), which is another ACLR method, regarding post-operative isokinetic and single leg hop test findings reported that PTA increased the functional recovery period and the RTS duration and that QTA and ST/G could be more beneficial for athletes [25,26,27,28]. The findings of a systematic review and meta-analysis study examining the effects of all graft types on isokinetic knee strength indicated weakness in extension strength in QTA and flexion strength in STG. It showed that PTA delayed recovery duration but had similar 1-year findings with the other two graft types regarding both extension and flexion strength [9]. Kim et al., in a meta-analysis study, reviewed numerous studies conducted on the isokinetic strength changes after ACL injuries and concluded that ACL injury caused loss of strength in both Ex and Flx phases, that the loss of strength in the Q muscle was approximately 3 times greater than that in the H muscle, and that these strength decreases caused a slight increase in the H/Q ratios [29]. In view of the results of Kim et al. and other researchers, we recommend the ST/G method to prevent ACL injury and the relatively high loss of strength in the Q muscle and to shorten the recovery period. Finally, it is a fact that the strength losses occurring after ACLR in all graft types will inhibit extensor and flexor strength due to deformation in the short term. Therefore, in the post-ACLR rehabilitation process, specific high-speed Ex and Flx exercises can be recommended to strengthen the ligament and to shorten RTS duration in order to prevent tibial anterolateral subluxations and painful symptoms concerning the ACLR side.

Unlike other studies, the present study examined the JAPT, TPT, and RD values in addition to PT and H/Q ratios obtained from isokinetic tests in athletes who underwent ST/G ACLR. Researchers have suggested that the JAPT value, which functions as a marker of the relationship between muscle length and strain, is an indicator of the risk of muscle injuries [30,31]. In the present study, our 6-month post-operative findings did not reveal any significance in JAPT values between the HK and ACLR sides at any angular velocity. Our findings imply that the risk of injury occurring as a result of muscle length and tension subsides within 6 months after ST/G ACLR. At this point, the dominant or non-dominant statuses of the HK, which served only as control, and ACLR sides prevented us from having a clear idea about this issue. A rapid muscle contraction capacity is required to stabilize sudden movements in the joints. [15,32]. Therefore, parameters related to muscle reaction time such as TPT and RD are also of great importance in determining joint injury risks [33,34]. While no significance was observed in TPT values at any of the angular velocities in our study, reciprocal delays were observed only at 60 and 180°/s Flx phases in RD. There are no isokinetic evaluations after ACLR in the literature examining TPT and RD parameters. However, researchers reported that TPT value may reveal different results in repeated measurements; therefore, care should be taken when making an evaluation [35]. Contrary to this view, there are also researchers who argue that TPT results are similar in repeated measurements [36,37]. If TPT times are evaluated regularly, particularly in athletes after ACLR, recovery can be evaluated not only in terms of maximum strength production but also in terms of duration. In the present study, the reciprocal delays observed in the ACLR sides in the Flx phases compared to the HKs in RD values implied that this may result from the deformation in the semitendinosus and gracilis muscles taken from the hamstring muscle with the ST/G graft method. 

The present study had several limitations. The main limitation of the present study was the lack of a healthy control group. Moreover, isokinetic evaluations were made only in concentric contractions and eccentric–concentric contractions exhibiting the agonist–antagonist structure of the knee were not used. Only the findings for the ST/G method were presented and relevant comments were made about other graft types. Finally, our research was designed for male athletes only. Inclusion of female athletes in future research and research in specific branches will contribute to the literature. 

## 5. Conclusions 

In conclusion, the findings of the present study showed that the 6-month post-operative isokinetic evaluations of the ST/G method revealed similar findings for the ACLR side and the HK side in a 6-month period in athletes; this can be used to make the decision to return to sports. However, we believe that future studies testing parameters such as JAPT, TPT, and RD with short-term repeated measurements will reveal better findings in terms of evaluating the post-operative ACLR results.

## Figures and Tables

**Table 1:** Descriptive data of the subjects.

	Mean	SD	Min.	Max.
Age (year)	24.65	7.47	18.00	35.00
Height (cm)	179.79	6.51	170.00	195.00
Weight (kg)	80.06	70.80	63.00	95.00
BMI (kg/m^2^)	24.80	3.43	20	32

SD, standard deviation; Min., minumum; Max., maximum.

**Table 2:** Comparison of pre-operative and post-operative levels of Lysholm, Tegner, and IKDC scores.

	Pre-op	Post-op	t	*p*	ES	%95 CI	
	Mean ± SD	Mean ± SD				LB	UB
Lysholm	71.96 ± 16.99	98.17 ± 3.45	−8.388	*p* < 0.001 *	0.40	−32.61	−19.81
IKDC	50.34 ± 8.58	90.97 ± 5.95	−23.13	*p* < 0.001 *	5.50	−44.21	−37.02
Tegner	6.48 ± 1.45	6.00 ± 1.64	0.701	*p* < 0.001 *	0.31	0.26	0.70

* *p* < 0.001; ES, Cohen’s d effect size; %95 CI, confidence interval; LB, lower bound; UB, upper bound.

## Data Availability

The datasets used and/or analyzed during the current study are available from the corresponding author on reasonable request.
